# Parasitaemia and Its Relation to Hematological Parameters and Liver Function among Patients Malaria in Abs, Hajjah, Northwest Yemen

**DOI:** 10.1155/2016/5954394

**Published:** 2016-03-09

**Authors:** Mohamed Al-Salahy, Bushra Shnawa, Gamal Abed, Ahmed Mandour, Ali Al-Ezzi

**Affiliations:** ^1^Zoology Department, Faculty of Science, Assiut University, Assiut 71516, Egypt; ^2^Biology Department, Faculty of Science, Soran University, Kurdistan, Iraq; ^3^Parasitology Department, Faculty of Medicine, Assiut University, Assiut 71516, Egypt; ^4^Biology Department, Faculty of Education, Aden University, Aden, Yemen

## Abstract

*Plasmodium falciparum* malaria is the most common infection in Yemen. The present study aims to investigate changes in hematological and hepatic function indices of* P. falciparum* infected individuals. This study included 67 suspected* falciparum* malarial patients attended in clinics and rural Abs Hospital (Tehama, Hajjah), Yemen, from October 2013 to April 2014. The diagnosis of malaria was confirmed by thick and thin film with Giemsa staining of malaria parasite. Hematological parameters and serum levels of aspartate transaminase (AST), alanine transaminase (ALT), alkaline phosphatase (ALP), and bilirubin (total and direct) as test indicators of liver function were studied. Patients with parasitaemia tended to have significantly lower hemoglobin, hematocrit, white blood cell count, lymphocytes, and platelets, compared with healthy normal subjects. Neutrophils levels were significantly higher in cases of* falciparum* malaria in comparison to healthy normal subjects. Serums AST, ALT, ALP, and bilirubin (total and direct) in* falciparum* malaria patients were significantly higher (*p* < 0.0001) than those of* falciparum* malaria of free individuals. Hematological and liver dysfunctions measured parameters were seen associated with moderate and severe parasitaemia infection. This study concludes that hematological and hepatic dysfunction parameters could be indicator of malaria in endemic regions.

## 1. Introduction

Malaria continues to be a great health problem in some of the most populated areas of the world. Infection is caused by a parasite of genus* Plasmodium* which is transmitted to human beings by infected female anopheles mosquito [1]. In Yemen the population at risk of malaria constitutes 81% of the total population, with estimated one million cases in 2009 [2].* P. falciparum* is the predominant species in Yemen where it is responsible for more than 90% of the malaria cases, with only minimal cases caused by* Plasmodium vivax* [3]. Studies have revealed that hematologic and biochemical changes occur in malaria infected blood and there are common complications associated with this disease. Hematological changes that are associated with malaria infection include anemia, thrombocytopenia, and disseminated intravascular coagulation [4–6]. Changes in physicochemical parameters of* P. falciparum* infested blood may vary with level of malaria endemicity, presence of haemoglobinopathies, nutritional status, demographic factors, and level of malaria immunity [7, 8]. The infection of liver cells by the sporozoites form of the malarial parasite can cause organ congestion, sinusoidal blockage, and cellular inflammation. These changes in hepatocytes can lead to the leakage of parenchymal (transaminases) and membranous (alkaline phosphatase) enzymes of the liver to the circulation. Hence increase in liver enzymes AST, ALT, and ALP observed in malaria infected patients also demonstrated that the serum activities of these liver enzymes increased with the increase in malarial parasite density. This change could confirm that the hepatic stage of the parasite's life cycle in human host is accompanied by significant perturbation in the hepatocyte's parenchyma and membrane leading to leakage of liver enzymes into the general circulation [9, 10].

Liver involvement in malaria is common in patients of severe malaria and may manifest as jaundice, hepatomegaly, and elevated liver enzymes like aspartate and alanine transaminases [11]. Hyperbilirubinemia, mainly unconjugated, is a common feature of* falciparum* malaria and is attributed to hemolysis of both parasitized and nonparasitized erythrocytes and partly due to liver damage [12]. Therefore, well-informed changes in blood parameters in malaria infection and hepatic function enable the clinician to establish reliable diagnosis and therapeutic interventions. Hence this research attempts to evaluate the changes in both hematological parameters and liver functions and in addition to evaluate the relationship between the levels of parasite density in blood and changes in hematological and hepatic function as biomarkers in* falciparum* malaria infected patients in Abs region of Yemen, which suffers from a highly malaria endemic.

## 2. Materials and Methods

### 2.1. Selection of Subjects

A total of 67 patients with malaria parasitaemia attending the clinics and Rural Hospital of Abs (Tehama, Hajjah), Yemen, were enrolled as the test subjects, who were reported ill with fever (temperature > 37.5°C), headache, vomiting, chills, diarrhea, and other clinical signs. Patients of age 18–48 years were selected who confirmed to be infected with the* falciparum* malarial parasite by microscopic examination of Giemsa stained thin blood smears. Patient's selection was done by simple random sampling of males and females between October 2013 and April 2014. 30 normal subjects free from malaria were used as control. Patients with hepatitis, smokers, and those who are taking any antimalaria drugs were excluded from this study.

### 2.2. Malarial Parasite Density Determination

*P. falciparum* parasitaemia was determined in various blood smears stained by Giemsa stain. Parasitaemia was calculated based on WHO [13]: low (+) 1–10/100 fields, mild (++) 11–100/100 fields, moderate (+++) 1–10/one field, and high parasitaemia (++++) >10/one field.

### 2.3. Sample Collection and Preparation

Sample collection and preparation: Venous blood was collected aseptically from the subjects using 5 mL disposable syringes. The blood samples were collected and 4 mL was transferred into plain bottles for the biochemical assays whereas the remaining 1 mL was transferred into EDTA bottles for malaria parasite tests. The blood samples in the plain bottles were allowed to clot and retract after which they were centrifuged at 3000 rpm for 10 min and the serum was transferred into sterilized plain bottles for the biochemical analysis.

### 2.4. Hematological Analysis

Whole blood samples were collected in EDTA tube for determination of hematological parameters including hemoglobin (Hb) concentration, tWBC count, neutrophils, lymphocytes, platelets, and packed cell volume (PCV) using automated (SYSMX.KX-21n) hematology analyzer.

### 2.5. Assays of Liver Functions Parameters

Serum aspartate transaminase and alanine transaminase (ALT) parameters were determined using Roche/Hitachi cobas c systems automatically. This assay follows the recommendations of the IFCC but was optimized for performance and stability by [14, 15]. Alkaline phosphatase (ALP) parameter was determined using Roche/Hitachi cobas c systems automatically by colorimetric assay in accordance with a standardized method. In the presence of magnesium and zinc ions, p-nitrophenyl phosphate is cleaved by phosphatases into phosphate and p-nitrophenol [16].

Serum bilirubin concentration was determined using Roche/Hitachi cobas c systems automatically and total bilirubin was determined by Diazo method [17], while direct bilirubin was determined by Diazo method (special) [18].

### 2.6. Statistical Analysis

The data were expressed as mean ± SE. The results were analyzed statistically using column statistics and one *t*-test. Correlation among the investigated parameters was tested by curves and regression using linear regression to test departure from linearity with runs test. These analyses were carried out using computer statistics Prism 3.0 Package (Graph and Software, Inc., San Diego, USA). The minimum level of statistical significance was set at *p* < 0.05, 0.01, or 0.001.

## 3. Result

Of the 67 patients infected with* P. falciparum* 11 (16.4%) had low intensity of infecting low 1+ (1–10/100 fields), 14 (20.9%) had mild intensity of infection 2+ (11–100/100 fields), 23 (34.3%) had moderate intensity of infection 3+ (1–10/one field), and 19 (28.4%) had high intensity of infection 4+ (>10/one field) (Table 1). There were significant decreases in the mean values of the hemoglobin (Hb), packed cell volume (PCV), total leucocyte counts (tWBC), lymphocytes, and platelets. Neutrophils were significantly higher in patients with* falciparum* malaria in comparison to healthy subjects (Table 2).  Table 3 shows the relationship between parasite density and hemoglobin and platelets.  Table 4 shows the changes in liver function biomarkers of the patient's* P. falciparum* malaria and healthy subjects. There were significant increases in the mean activity enzymes values of the AST, ALT, ALP, and serum total and direct bilirubin.  Table 5 shows the relationship between parasite density and liver enzymes activities in serum. Level of parasitaemia correlates positively with mean liver enzyme activities specifically moderate and high parasitaemia showing higher values of liver enzyme activities when compared with patients having low and mild parasitaemia. Total bilirubin and direct bilirubin showed increase with increase in severity of parasitaemia of malarial infections (Table 6).

## 4. Discussion

In this study, hemoglobin (Hb) and packed cell volume (PCV) decreased in* P. falciparum* affected patients compared to the healthy subjects. This finding agrees with previous reports [5, 19]. The earlier studies reported a significant reduction in hemoglobin concentration and packed cell volume in patients with malaria parasitaemia. In this study the mean hemoglobin concentration showed 9.3 g/dL, but, in the study done by Nadeem et al. [20], hemoglobin level in* P. falciparum* affected patients was 13.7 gm/dL. This value was more than that observed in our study. Reduced hemoglobin in malaria may be attributed to the increase of breakdown red blood cells by the parasites [21]. According to the report of Maina et al., [5] as contained in the National Guidelines for Diagnosis Treatment and Prevention of Malaria for Health Workers in Kenya, anemia is defined as [Hb] < 10 g/dL for both males and females. Furthermore, severe malarial anemia is defined as [Hb] < 5.5 g/dL. Therefore, the drop in hemoglobin concentrations in the malarious subjects in our study ranged between 5 and 10.5 g/dL, ranging approximately from mild to moderate anemia. In study done by Bakhubaira [22], it was observed that anemia is more common amongst the patients infected by* P. falciparum* in Aden, Yemen.

Lower mean value for leukocyte count in the* falciparum* malaria patients compared to the healthy subjects was observed in our study. This was in concordance with other studies [23]. Similarly, decrease in WBC in malaria patients has been observed by S. Chandra and H. Chandra [24], who reported that low leukocyte count may be used as probable indicator for malaria in endemic countries. The present study reports a significant reduction in lymphocytes level in individuals infected with* P. falciparum* as compared to those not infected (healthy subjects). Reduction in the counts of lymphocytes was observed in some studies [25]. The decrease in lymphocyte counts associated with malaria may be due to reflecting redistribution of lymphocytes with sequestration in the spleen [26].

The present study reports a significant increase in neutrophil level of individuals infected with* P. falciparum* as compared to those not infected. These findings are in agreement with previous reports [5, 25]. Increase of neutrophil in these cases could be a representation of early release of neutrophil from the bone in response to the infection. In our study, thrombocytopenia emerged as a predictor of malaria which is an observation of many studies which confirm it [19, 27, 28]. Low platelet count is a finding of malarial infection and thrombocytopenia may be more common than anemia in malaria infection.

The results reported in this study show some significant increases in activities enzymes aspartate transaminase (AST), alanine transaminase (ALT), and alkaline phosphatase (ALP) among patients with* P. falciparum* malaria. The increased serum levels of hepatic enzymes, transaminases (AST and ALT), and ALP are the biomarkers of liver disorders. Our results are consistent with other studies which reported that majority of the patients show elevation in serum activities (AST, ALT, and ALP) indicating liver damage [29, 30]. The increases in serum level of hepatic enzymes, transaminases (SGOT and SGPT), and alkaline phosphatase are the markers of liver damage. Ignatius et al. [31] reported that the liver enzymes leakage and bilirubin increased with increase in malaria parasite density. Usually, in uncomplicated malaria, raised bilirubin is mainly due to hemolysis of parasitized and nonparasitized RBC and/or hepatocytes damage [32]. In this study raised bilirubin was mainly due to hepatic dysfunction. Molyneux et al. [33] suggested that jaundice, which may be deep, is usually accompanied by only moderate elevation of hepatic enzymes and results more from hemolysis than from hepatic damage. In the present study, the cause of jaundice was attributed mainly to intravascular hemolysis and hepatic dysfunction (42.1%), as reported by other studies [34, 35].

## 5. Conclusion

Hemoglobin, platelets count, lymphopenia, tWBC, and neutrophilia were seen associated with mild, moderate, and severe parasitaemia infection. Acute* falciparum* malaria infection is associated with an increase in serum activity of aspartate and alanine aminotransferases and alkaline phosphatase thus indicating that the infection is associated with acute liver injury.

## Figures and Tables

**Table 1:** Occurrence of *P. falciparum* malaria in parasitaemia patients by intensity of infections.

Intensity of infection (Parasitaemia) *N* = 67	Number of patients with *falciparum* malaria (%)
Low (+)	11 (16.4)
Mild (++)	14 (20.9)
Moderate (+++)	23 (34.3)
High (++++)	19 (28.4)

**Table 2:** Comparison of hematological parameters between the cases of *P. falciparum* malaria infection and healthy subjects.

Parameters	Patients with malaria	Healthy control subjects
	*N* = 67	*N* = 30
Hb (g/dL)	9.4 ± 0.28^*∗∗∗*^	13.08 ± 0.36
PCV (%)	29.47 ± 0.82^*∗∗∗*^	42.52 ± 0.86
T.WBC (10^3^/uL)	5.91 ± 0.35^*∗∗*^	7.95 ± 0.60
Lymphocytes (%)	24.64 ± 1.63^*∗∗∗*^	39.17 ± 2.19
Neutrophils (%)	71.28 ± 1.60^*∗∗∗*^	48.46 ± 2.30
Platelet (×10^3^/*µ*L)	116.6 ± 7.03^*∗∗∗*^	353.4 ± 18.72

^*∗∗*^ *p* < 0.01; ^*∗∗∗*^ *p* < 0.0001.

**Table 3:** The level of hemoglobin and platelets in patient *P. falciparum *malaria with parasitaemia.

Parasitaemia/field	Mean hemoglobin	Mean platelets
Low (+)	12.75 ± 0.2	177.5 ± 5.1^*∗∗∗*^
Mild (++)	10.95 ± 0.1^*∗∗∗*^	131.5 ± 7.8^*∗∗∗*^
Moderate (+++)	8.64 ± 0.3^*∗∗∗*^	83.8 ± 2.6^*∗∗∗*^
High (++++)	8.14 ± 0.4^*∗∗∗*^	53.1 ± 6.9^*∗∗∗*^
Healthy	13.08 ± 0.3	353.4 ± 18.7

*∗∗∗* = *p* < 0.0001.

**Table 4:** Changes in liver functions biomarkers of the patient's *P. falciparum* malaria and healthy subjects.

Parameters	Patients with malaria	Healthy subjects
	*N* = 67	*N* = 30
Aspartate aminotransferase (AST) U/L	51.12 ± 2.85^*∗∗∗*^	29.20 ± 1.50
Alanine aminotransferase (ALT) U/L	37.92 ± 2.04^*∗∗∗*^	20.97 ± 1.42
Alkaline phosphatase (ALP) U/L	177.4 ± 10.49^*∗∗*^	108.2 ± 6.81
Total bilirubin (TB) umol/L	70.13 ± 7.04^*∗∗∗*^	6.15 ± 0.39
Direct bilirubin (DB) umol/L	14.61 ± 1.78^*∗∗∗*^	2.05 ± 0.18

^*∗∗*^ *p* < 0.01; ^*∗∗∗*^ *p* < 0.0001.

**Table 5:** Mean liver enzymes levels in parasitaemia *P. falciparum* malaria and healthy control subjects.

Mean parasitaemia/field	AST level (U/L)	ALT level (U/L)	ALP level (U/L)
Low (+)	28.36 ± 1.04	22.52 ± 1.52	95.69 ± 4.88
Mild (+)	34.26 ± 1.45^*∗*^	25.89 ± 2.34	137.6 ± 9.73^*∗*^
Moderate (+)	58.63 ± 3.17^*∗∗∗*^	47.27 ± 1.73^*∗∗∗*^	194.0 ± 15.06^*∗∗∗*^
High (+)	96.03 ± 6.21^*∗∗∗*^	60.28 ± 5.58^*∗∗∗*^	228.5 ± 23.97^*∗∗∗*^
Healthy subjects	29.24 ± 1.04	20.90 ± 0.98	108.2 ± 4.80

^*∗*^ *p* < 0.01; ^*∗∗∗*^ *p* < 0.000.

**Table 6:** Mean total and direct bilirubin levels in parasitaemia *P. falciparum* malaria and healthy control subjects.

Parasitaemia	TB level (umol/L)	DB level (umol/L)
Low (+)	8.54 ± 0.68^*∗∗*^	2.48 ± 0.16
Mild (+)	19.24 ± 1.46^*∗∗∗*^	5.27 ± 0.35^*∗∗∗*^
Moderate (+)	68.31 ± 6.45^*∗∗∗*^	23.53 ± 4.13^*∗∗∗*^
High (+)	137.6 ± 13.25^*∗∗∗*^	31.82 ± 3.88^*∗∗∗*^
Healthy subjects	6.12 ± 0.27	3.33 ± 0.26

^*∗∗*^ *p* < 0.001; ^*∗∗∗*^ *p* < 0.0001.
